# Whole genome sequencing of multidrug-resistant *Mycobacterium tuberculosis* isolates collected in the Czech Republic, 2005–2020

**DOI:** 10.1038/s41598-022-11287-5

**Published:** 2022-05-03

**Authors:** Matúš Dohál, Věra Dvořáková, Miluše Šperková, Martina Pinková, Andrea Spitaleri, Anders Norman, Andrea Maurizio Cabibbe, Erik Michael Rasmussen, Igor Porvazník, Mária Škereňová, Ivan Solovič, Daniela Maria Cirillo, Juraj Mokrý

**Affiliations:** 1 Department of Pharmacology and Biomedical Centre Martin, Jessenius Faculty of Medicine in Martin, Comenius University, Bratislava, Slovakia; 2 National Reference Laboratory for Mycobacteria, National Institute of Public Health, Praha, Czech Republic; 3 Emerging Bacterial Pathogens Unit, IRCCS San Raffaele Scientific Institute, Milan, Italy; 4 International Reference Laboratory of Mycobacteriology, Statens Serum Institut, Copenhagen, Denmark; 5 National Institute of Tuberculosis, Lung Diseases and Thoracic Surgery, Vyšné Hágy, Slovakia; 6 Faculty of Health, Catholic University, Ružomberok, Slovakia; 7 Department of Molecular Medicine and Biomedical Centre Martin, Jessenius Faculty of Medicine in Martin, Comenius University, Bratislava, Slovakia; 8 Department of Clinical Biochemistry, Jessenius Faculty of Medicine in Martin, Comenius University, Bratislava, Slovakia

**Keywords:** Infectious-disease diagnostics, Clinical microbiology

## Abstract

The emergence and spread of resistant tuberculosis (TB) pose a threat to public health, so it is necessary to diagnose the drug-resistant forms in a clinically short time frame and closely monitor their transmission. In this study, we carried out a first whole genome sequencing (WGS)-based analysis of multidrug resistant (MDR) *M. tuberculosis* strains to explore the phylogenetic lineages diversity, drug resistance mechanisms, and ongoing transmission chains within the country. In total, 65 isolates phenotypically resistant to at least rifampicin and isoniazid collected in the Czech Republic in 2005–2020 were enrolled for further analysis. The agreement of the results obtained by WGS with phenotypic drug susceptibility testing (pDST) in the determination of resistance to isoniazid, rifampicin, pyrazinamide, streptomycin, second-line injectables and fluoroquinolones was more than 80%. Phylogenetic analysis of WGS data revealed that the majority of MDR *M. tuberculosis* isolates were the Beijing lineage 2.2.1 (n = 46/65; 70.8%), while the remaining strains belonged to Euro-American lineage. Cluster analysis with a predefined cut-off distance of less than 12 single nucleotide polymorphisms between isolates showed 19 isolates in 6 clusters (clustering rate 29.2%), located mainly in the region of the capital city of Prague. This study highlights the utility of WGS as a high-resolution approach in the diagnosis, characterization of resistance patterns, and molecular-epidemiological analysis of resistant TB in the country.

## Introduction

Tuberculosis (TB), an infectious disease caused by the *Mycobacterium tuberculosis* complex (MTBC), is still a public health problem and its treatment is challenging for clinicians. Case notifications of people newly diagnosed with TB has a declining trend, but the number of deaths rose to 1.5 million in 2020, which is probably related to the COVID-19 pandemic^1^. The main problem complicating the eradication and treatment of TB is the prevalence of resistant strains, predominantly multidrug resistant (MDR-TB, resistant to 2 first-line drugs: isoniazid and rifampicin) and extensively-drug resistant (XDR-TB, resistant to fluoroquinolones and injectable drugs in addition to MDR)^1^. In June 2021, the World Health Organization (WHO) adopted a new definition of XDR-TB as TB caused by *M. tuberculosis* strains that are resistant to isoniazid, rifampicin, any fluoroquinolone, and either bedaquiline or linezolid (or both)^2^. However, in our study, we followed the original definition of XDR, as the data is before 2021.

**Figure 1:**
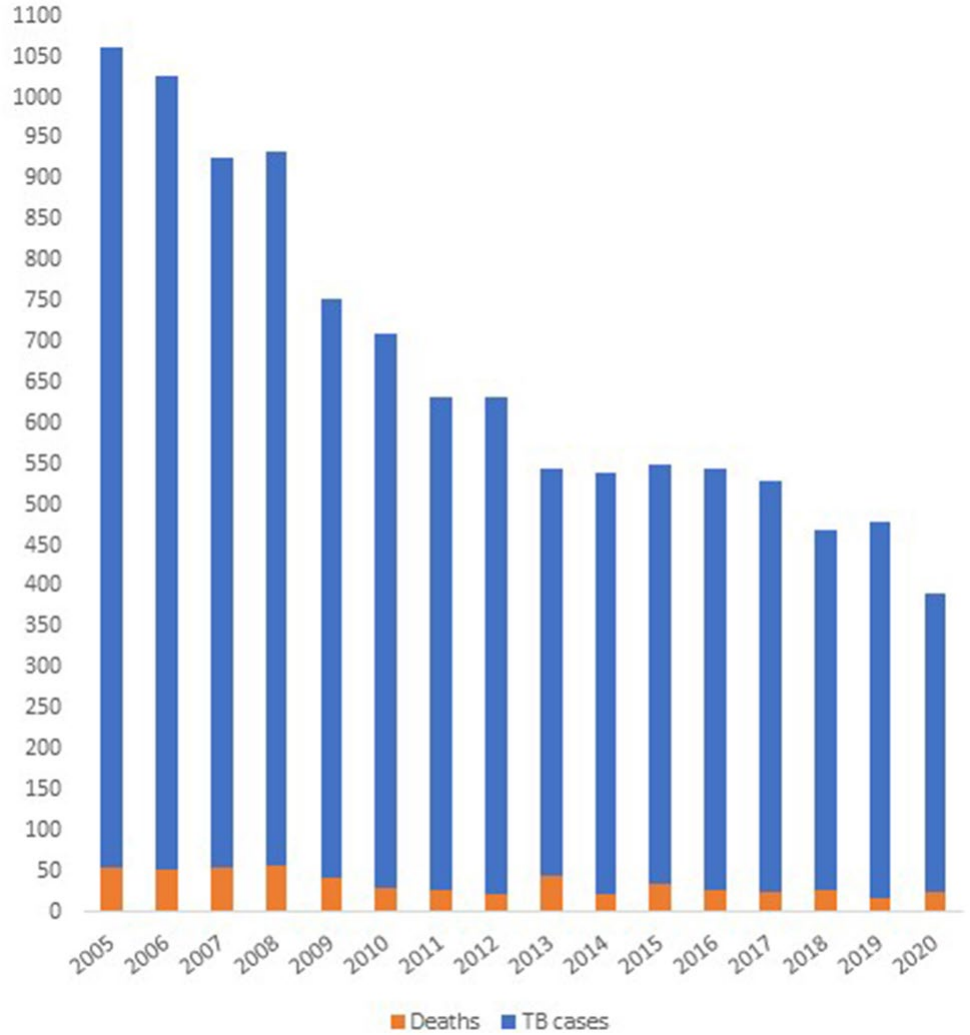
Incidence of tuberculosis and number of deaths during 2005–2020 in the Czech Republic.

From 2005 to 2020, the incidence of TB as well as the related deaths decreased significantly in the Czech Republic, so it is currently considered a country with a low incidence of TB (Fig. 1).

Phenotypic drug susceptibility testing (pDST) is routinely performed on all TB culture-positive specimens in the country. The results from 2005 to 2020 showed a total of 480 strains of *M. tuberculosis* resistant to at least one antituberculosis drug. More specifically, in 2005–2020, the estimated proportion of MDR and XDR strains was 2.13% of all phenotypically tested isolates (of which 17.4% in previously treated patients)^3^.

Culture-based pDST on solid or liquid (*Mycobacterium* growth indicator tubes—MGIT) media and genotypic drug susceptibility testing (gDST) methods (such as Xpert MTB/RIF, Cepheid, Sunnyvale, California, USA and GenoType MTBDR, Hain LifeScience GmbH, Nehren, Germany) are currently preferred for the determination of *M. tuberculosis* resistance in clinical settings^4^. Limitations of pDST and gDST lead to the development and application of advanced molecular techniques such as whole genome sequencing (WGS). WGS data provide a rapid and detailed insight of mutations encoding resistance to all available antituberculosis drugs and contribute to the identification of the transmission patterns based on the genomic relatedness of *M. tuberculosis* strains, without the need for traditional contact tracing^5–8^. The molecular-epidemiological analysis of TB in the Czech Republic was conducted by so-called classical typing methods^9^. However, these methods interrogate only a small fraction of the *M. tuberculosis* genome to reconstruct the complex transmission chains and do not show sufficient discriminatory power to track the transmission links between patients^10^.

Despite the fact that a large proportion (more than 28%) of patients diagnosed with TB are born outside the Czech Republic, the genetic relatedness of drug-resistant *M. tuberculosis* strains has not yet been studied in detail^11^. The aim of this work was to perform the first WGS-based study using the MDR-, XDR-isolates collected in the Czech Republic during the years 2005–2020. Sequencing analysis determined the complete resistance profile and revealed the phylogenetic diversity of the strains, thus contributing to the characterization of local outbreaks and the monitoring of the dynamics of resistant strains over the years. This work will serve as a baseline to analyse the molecular epidemiology and drug-resistance of *M. tuberculosis* strains in the Czech Republic.

## Results

### Sample collection

A total of 65 samples of MDR *M. tuberculosis* strains were included, representing 60% of the total number of MDR-TB cases diagnosed during the study. A limited number of samples were due to the inability to reactivate some cultures in MGIT medium. Samples have been collected continuously over the years, specifically: 3 in 2005, 1 in 2006, 3 in 2008, 7 in 2009, 2 in 2010, 4 in 2014, 9 in 2015, 4 in 2016, 9 in 2017, 10 in 2018, 12 in 2019 and 1 in 2020. Subsequent isolates from the same TB patient were included in the study (only in two cases: CZ800-17/CZ541-18 and CZ628-17/CZ149-18) as long as the isolation date is more than six months after the isolation date of the previous isolate. The study included 14 women and 47 men with a mean age of 40 years (ranging from 23 to 81 years).

### Sequencing data quality

The minimum criteria for sequencing data quality were: mean coverage ≥ 30 and mapped reads ≥ 90% of the reference genome covered by the sequence read. These requirements were met for all samples, as the average depth of coverage was 90× (ranging from 39× to 158×) and mapped reads 98.59% (ranging from 94.71 to 99.33%).

### Drug susceptibility testing

Phenotypic testing confirmed resistance to rifampicin and isoniazid (i.e. MDR-TB) in all 65 samples. Moreover, resistance to at least one additional first-line or second-line antituberculosis drug was determined in 64 samples (98.46%, Table 1).

**Table 1:** Overview of additional phenotypic resistance of MDR strains to first-line and second-line antituberculosis drugs.

Resistance	Number	%
MDR	65	100
+ EMB	35	50.77
+ PZA	21	24.62
+ STM	60	93.85
+ ETO	25	30.77
+ AMG	15	18.46
+ FQ	18	16.92
+ PAS	13	16.92
+ CS	6	9.2

*EMB* ethambutol, *PZA* pyrazinamide, *STM* streptomycin, *AMG* aminoglycosides (kanamycin, amikacin, capreomycin), *ETO* ethionamide, *FQ* fluoroquinolones, *PAS* para aminosalicylic acid, *CS* cycloserine.

Sequencing data revealed a wide range of gene mutations associated with resistance to first-line and second-line antituberculosis drugs, including delamanide (Table 2). Moreover, resistance to rifampicin was primarily caused by the mutations at codon 450 (S450L) in the *rpoB* gene (56/65 resistant isolates)^12^. In addition, mutations at codon 435 (A435V; A435T; 4 patients), 445 (H445L; 2 patients) and 452 (L452P; 1 patient) were also characterized in the *rpoB* gene. An additional mutation in the *rpoC* gene (L527V; P452S; I491T) was identified in 11 samples (16.92%)^12^. Compensatory mutations in the *rpoC* gene have been primarily associated with the *rpoB* S450L mutation and Euro-American sublineage 4.1.2.1, which is in concordance with other studies examining the significance of these mutations^13^. Deletion in the *rpoB* gene (1302_1307delGGACCA) was detected in one phenotypic resistant isolate (CZ412-18). Isoniazid-resistant isolates most frequently showed mutations in the *katG* gene (S315T, 64/65). A mutation in the *inhA* gene (S94A) was found in one isolate. Sixteen isolates (24.62%) showed simultaneous mutations in the *fabG1* gene promoter region (15C>T, 14/65; 8T>C, 2/65)^14^. Fifty-seven ethambutol-resistant isolates harboured mutations in the *embB* (primarily M306I/ M306L, 27/57; followed by G497A; A435A; T334H; T319C; G406A; S297A; P404S) and *embA* (including c.-16C>T, c.-12C>T; 5/57) genes^15,16^. Mutations encoding resistance to ethambutol were also identified in 23 phenotypically-sensitive isolates, which is the main reason for the low reliability (sensitivity and specificity 97.22% and 17.24%, respectively) of pDST for ethambutol. Compared to other antituberculosis drugs, the greatest mutational variability was observed in *pncA* gene encoding the resistance to pyrazinamide. Twenty-two different non-synonymous mutations and 2 novel deletion polymorphisms (287_287delA; 524_524delT) in *pncA* gene have been identified with 95% sensitivity and 73% specificity (Table 2, Supplementary Table 2)^17^. The most frequent were mutations at codons 63 (3/37) and 160 (3/37). Genotypic resistance to streptomycin was confirmed in 58/65 (87.7%) (sensitivity 93.33% and specificity 80%). Mutations in the *rpsL* (predominantly L43A—47/58 and L88A—7/58) and *rrs* (514a>c; 11/58) genes were most common^18^. Resistance across the second-line injections (kanamycin, amikacin, capreomycin) was induced by mutations occurring in the *rrs* (1401a>g), *eis* (10g>a; 12c>t) and *tlyA* (733_742delACGCAGACCG) genes and detected in 21 isolates (32.31%)^19^. Of these, 10 isolates (47.62%) were resistant to all second-line injectables, 10 isolates (47.62%) resistant to kanamycin and one isolate (4.61%) resistant to kanamycin simultaneously with capreomycin. Regarding fluoroquinolone resistance, the 17 isolates (26.15%) harboured various mutations only in the *gyrA* gene (A90V; A94G; A94A; S91P)^20^. In overall, the resistance to second-line injectables and fluoroquinolones was determined with a sensitivity and specificity of 80% / 93.75% and 82.22%/93.88%, respectively.

**Table 2:** List of mutations in the corresponding genes encoding resistance to first- and second-line antituberculosis drugs.

Drug	Gene	Mutation
Rifampicin	*rpoB*	S450L; H445L; A435V; A435T; L452P; c.1302_1307del GGACCA
Isoniazid	*katG*	S315T
	*inhA*	S94A
	*fabG/fabG1*	15C>T; 8C>T
Ethambutol	*embB*	M306I; M306L; G497A; A435A; T334H; T319C; G406A; S297A; P404S
	*embA*	16C>T; 12C>T
Pyrazinamide	*pncA*	T142A; T160A; G10L; H71A; 405_406insCACC; H51A; C138T; A63A; H57A; L27P; P94L; P69L; P63G; V139A; A12A; I133T; I31S; V128G; T34STOP; T68G; L19P; A49G; G97A; *287_287delA; *524_524delT
Streptomycin	*rpsL*	L43A; L88A
	*rrs*	514a>c; 513c<t; 906a>g
Second-line injectables	*rrs*	1401a>g
	*eis*	12C>T; 10G>A
	*tlyA*	c.733_742del
Fluoroquinolones	*gyrA*	A90V; A94G; A94A; S91P
Ethionamide	*fabG*	15C>T; 8T>C
	*ethA*	110_110del; 1054_1054del; T314I; M1A; 4327275_4327547del; 1010_1010del; 341_341del; 32_32del; 1386_1386del
	*inhA*	S94A
Para-aminosalicylic acid	*folC*	A49P; G40G
Linezolid	*rplC*	C154A
Delamanid	*ddn*	T88STOP

*Novel mutation—not yet reported in the literature.

In addition, mutations associated with resistance to other second-line antituberculosis drugs have been identified, resulting in: 33 isolates (50.77%) resistant to ethionamide (mutation in *ethA* gene: 110_110delT; 1054_1054delC; T314I; M1A; A67P; 1010_1010delA; 341_341delT; 32_32delC; 1386_1386delT; mutation in *fabG*1 gene: 15C>T; 8C>T) (sensitivity 72% and specificity 60%), 3 isolates (4.61%) resistant to paraaminosalicylic acid (*folC* G40G; A49P), and 2 isolates (3.08%) resistant to linezolid (*rplC* C154A). Two isolates (3.08%) showed gene mutations in the *ddn* gene (T88STOP) encoding delamanide resistance^21^. No mutations in the genes encoding resistance to bedaquiline and clofazimine have been identified.

In overall, 10 isolates were determined as XDR.

### Phylogenetic analysis and identification of transmission cluster

**Figure 2:**
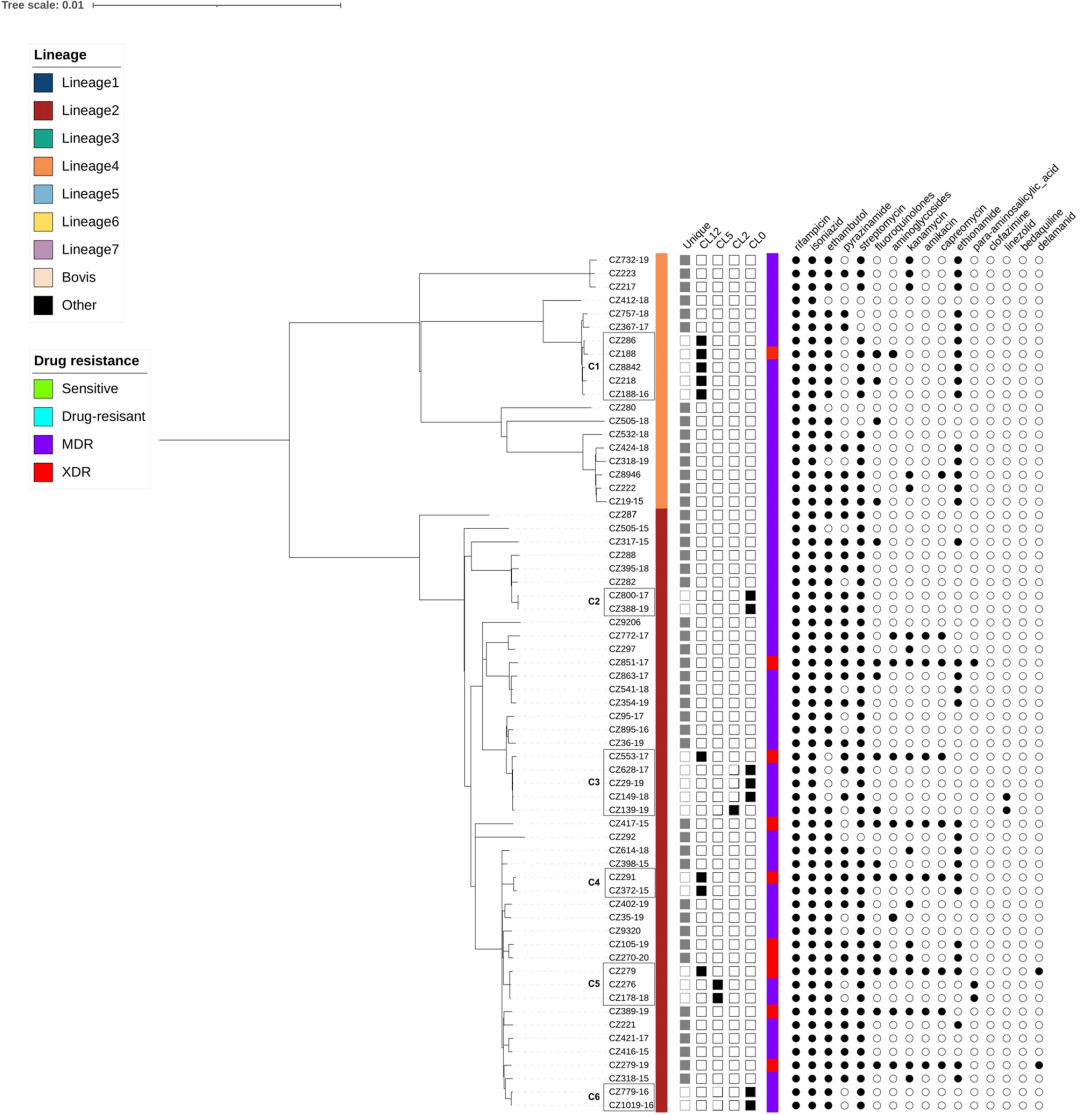
Maximum likelihood phylogenetic tree generated from 64 MDR isolates collected in the Czech Republic during the years 2005–2020 together with their lineage, cluster assignment, and genotypic resistance to first- and second-line antituberculosis drugs. The tree was annotated using iTOL v6 (https://itol.embl.de/). The first column denotes the lineages. The next 5 columns show genomic relatedness within clusters (CL12, CL5, CL2 and CL0—sets the maximum number of SNPs that differ from the genetically closest isolate). Mutations encoded resistance are represented by filled circles (presence of mutation) or empty circles (absence of mutation) icons. *MDR* multi-drug resistant, *XDR* extensively-drug resistant, drug-resistant (including mono- and poly-resistant).

Two major lineages were observed through the WGS analysis. Phylogenetic analysis showed that most isolates (46; 70.77%) belonged to lineage 2 (specifically to 2.2.1 Beijing genotype family). Twenty isolates (30.77%) were included in lineage 4. The classification of isolates into sublineages was as follows: 9 belonged to sublineage 4.1.2.1 (Haarlem), 6 to sublineage 4.3.3 (LAM, T), 3 to sublineage 4.2.1 (Ural) and 1 to sublineage 4.3.4.2 (LAM, T). One isolate (CZ445-15) consisted of a mix of 4.3.3 and 4.1.2.1 sublineages. Additional subclassification for strains belonging to the same sublineage can be seen in the maximum likelihood phylogenetic tree (Fig. 2).

**Figure 3:**
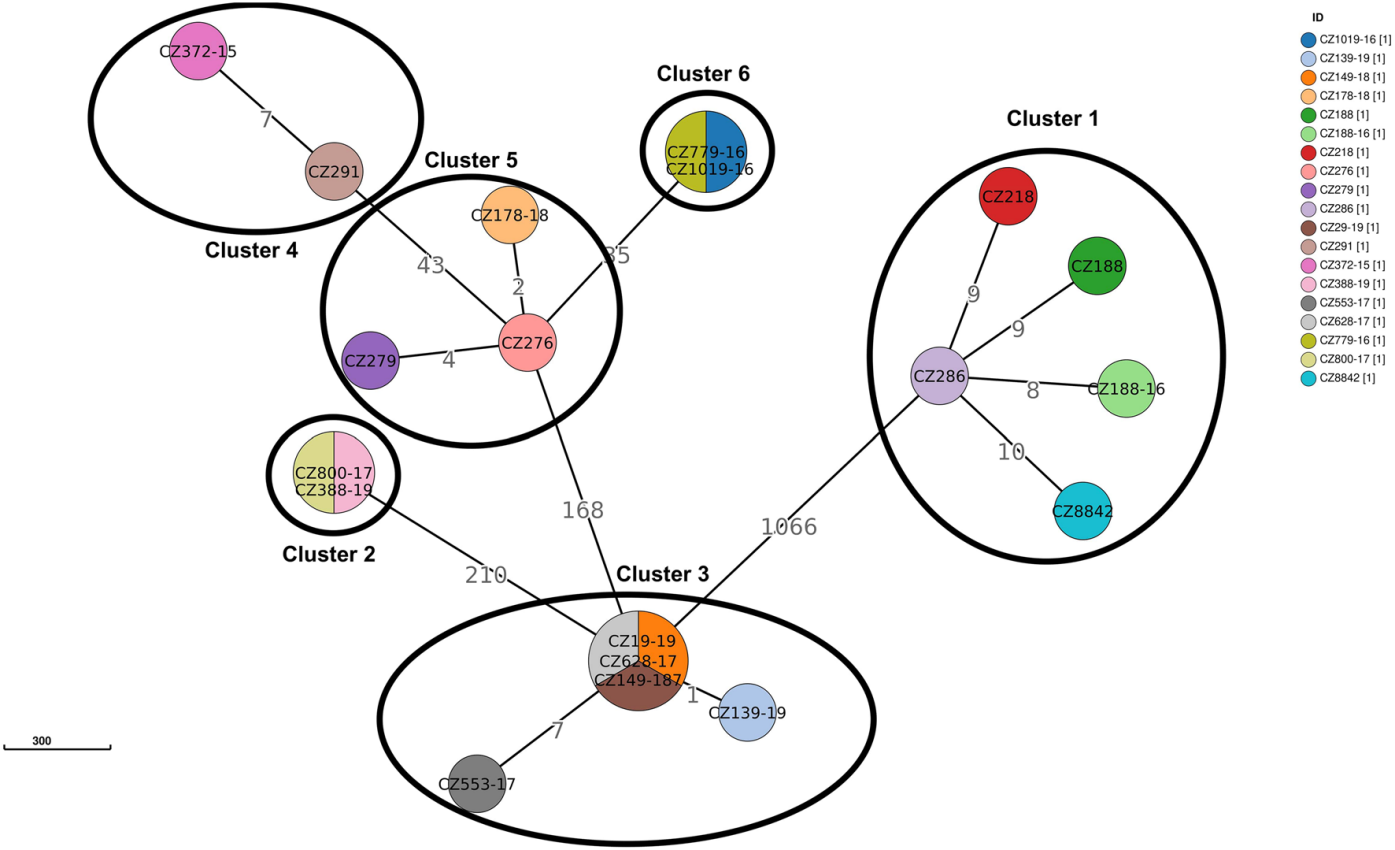
Minimum spanning tree based on SNP differences between the strains, including the XDR-TB and MDR-TB strains collected in Czech Republic during 2005 and 2020. Maximum distance set to 12 SNPs for linked transmission. Distant matrix generated from MTBseq (version 1.0.2, https://github.com/ngs-fzb/MTBseq_source) and a minimum spanning tree was constructed using GrapeTree software (https://achtman-lab.github.io/GrapeTree/MSTree_holder.html).

Cluster analysis with a predefined maximum cut-off of 12 SNPs difference revealed 6 clusters containing 15 MDR isolates and 4 XDR isolates (n = 19; clustering rate 29.23%) with a maximum size of 2–5 isolates for each cluster (Fig. 2). Nine isolates were obtained from patients with newly diagnosed TB and 10 from patients with confirmed recurrent TB. These clusters consisted mainly of strains from Beijing sublineage 2.2.1 (73.68%) and to a lesser extent from Euro-American sublineage 4.1.2.1 (Haarlem) (26.32%). A difference of 0–2 SNPs between patients was determined in 8 cases (Fig. 2, CL0, CL2), indicating a recent transmission events and the difference 3–5 SNPs was documented in only 2 patients, indicating the direct transmission (Fig. 2, CL5). Using a threshold 6–12 SNPs to look at older transmission events, 9 patients in 4 clusters were identified (Fig. 2, CL12). The distance between the individual clusters ranged from 35 SNPs (between cluster 5 and 6) to 1066 SNPs (between cluster 3 and 1) (Fig. 3).

Four isolates (80%) from cluster 1 come from the capital city of Prague. Patient CZ8842 is from another region (Nový Jičín) but was first diagnosed with MDR-TB in 2005 (as well as patients CZ188 and CZ218), suggesting an association with other isolates from this cluster. Epidemiological data on patients grouped in clusters 2 and 3 also indicated the spread of a resistant strain of *M. tuberculosis* from Prague region. Cluster 5 was formed by three isolates, two of which related to the same accommodation facility in the town of Šenov. The remaining clusters consisted of only two isolates that came from geographically separated areas (Supplementary Table 1).

## Discussion

WGS is a molecular-genetic method that is becoming increasingly preferred in clinical laboratories; however, it has not yet been performed on continuously collected MDR and XDR isolates. Therefore, we conducted the first WGS study in the country. Sequencing data in our study showed that in the last 15 years, MDR/XDR strains of *M. tuberculosis* belonging to the Beijing 2.2.1 lineage were the most common in the Czech Republic (70.77% of all isolates), followed by the Euro-American lineage 4 (29.23%). The results correlate with studies that have confirmed a growing incidence and transmission of MDR strains of this lineage in Eastern Europe, predominantly due to selective advantages including hypervirulence, hypermutability and increased transmissibility^22–25^. In comparison, the molecular-epidemiological study based on IS*6110*-RFLP conducted in Czech Republic in 2006 showed that only 7.1% of all isolates (11/155) belonged to the Beijing lineage, but represented up to 36% of all resistant isolates^9^. We assume that the prevalence of the Beijing lineage is evolutionary associated with migration events from Ukraine, which began in early 1990s. It reflects that the Ukrainian national minority is currently one of the largest membership bases in the Czech Republic^26^. In general, the prevalence of Beijing strains among MDR isolates is of growing concern, especially due to evolutionary success in terms of dispersion and adaptation to different human populations; therefore, appropriate measures should be taken for public health surveillance^27^.

In relation to resistance, XDR-TB was significantly more often detected in MDR-TB strains of the Beijing genotype (9/10; 90%) than in MDR-TB strains of non-Beijing genotypes (1/10; 10%). These data are supported by a study by Beer et al., which confirmed a significantly increased proportion of Beijing genotype (84%) among all XDR strains collected in the European Union^28^. In general, the most prevalent gene mutations reported in our study were *rpoB* S450L for rifampicin, *katG* S315T for isoniazid, *embB* M306I for ethambutol, *pncA* H57A for pyrazinamide, *rpsL* L43A for streptomycin, *gyrA* A94G for fluoroquinolones, *rrs* 1401a>g and consistent with other studies^29,30^. Moreover, pDST was performed on all samples; therefore, the identified gene variants can potentially contribute to increasing the sensitivity of currently used line-probe assays and developing new genotypic arrays. In comparison with the available catalogues summarizing the mutations associated with resistance, we identified two novel potential resistance-confirming indels in the *pncA gene* (287_287del; 524_524del)^29,31^. These mutations require further detailed investigation to determine their clinical relevance and impact on minimal inhibitory concentration to pyrazinamide. Additionally, we reported the first two strains of *M. tuberculosis* exhibiting genotypic resistance to new antituberculosis drug delamanid. The resistance was encoded by mutations in *ddn* gene. Interestingly, delamanid-resistant strains were isolated from patients without a prior treatment regimen containing this drug, probably due to a high rate of spontaneous mutations in the target gene^32^. These data are also supported by other studies that have confirmed resistance in patients without previous exposure to this drug^21,33^. Our results highlight the need to determine susceptibility to delamanid in clinical laboratories in order to prevent the spread and selection of delamanid-resistant strains.

Phylogenetic analysis of *M. tuberculosis* genomes from subsequent samples from the same patient confirmed reinfection with a different strain of *M. tuberculosis* (difference more than 12 SNPs) in patient 800-17/541-18 (man, 31/32 years old). The same analysis from isolates from patient 628-17/149-18 (man, 37/38 years old) showed a difference of 0 SNPs between strains. However, additional genotypic resistance to linezolid developed within 1 year (Fig. 2, Supplementary Table 2). We hypothesize that mutations associated with resistance to linezolid developed due to non-adherence to treatment regimen or incorrect combination and dosing of antituberculosis drugs^34^.

The overall sensitivities and specificities for WGS, as mentioned in the results (“Drug susceptibility testing” section), demonstrated more than 80% concordance of the results obtained by WGS with pDST in the determination of resistance to isoniazid, rifampicin, pyrazinamide, streptomycin, second-line injectables, and fluoroquinolones, which is consistent with other available studies. studies^14–37^. The lower specificity of WGS in ethambutol resistance is due to the genotypic resistance of 23 phenotypically sensitive isolates. These isolates showed mutations in the *embB* gene, especially at codons 306, 406 and 497. Mutations at these codons were also identified in phenotypically resistant isolates. This phenomenon is not uncommon and has been observed in other studies, as these mutations may only slightly increase the minimum inhibitory concentration and thus lead to false negative pDST results^38–40^.

After cluster analysis, six clusters containing a total of 19 strains were identified (clustering rate 29.23%), most of which were formed by Beijing lineage isolates. In general, the clustering rate in our study was lower compared to the continuous 20 years genotyping study of MDR-TB in Spain (clustering rate 48.4%), 4-years drug-resistant TB WGS study in Thailand (48.5%) and the 3-years study involving MDR isolates from European Union countries (clustering rate 51.6%)^41–43^. These findings confirm the discriminatory power of WGS in terms of the definition of clonal complexes and the diversity of genotypes circulating in the country and highlight the need for a public health service to control and diagnose resistant TB. Furthermore, the results suggest that we lack some isolates within the clusters, or that resistance has developed due to inappropriate TB treatment regimens, as well as patient non-compliance. The higher clustering rate exhibited by the Beijing lineage (31.81%) compared to the Euro-American lineage (25%) is consistent with previous recent studies^44,45^. Also, more than 50% of clustered isolates are located in the Prague region, the capital of the Czech Republic with the largest proportion of foreign population due to employment. The overall clustering rate (29.23%) was almost half lower compared to study using *Mycobacterium tuberculosis*-specific multiple locus variable number tandem repeat (MIRU-VNTR) method for evaluation the genetic relatedness of MDR isolates collected in the Czech Republic during the years 2003–2011^28^. This may be due to false clustering and lower resolution of transmission using MIRU-VNTR in countries with low TB incidence^46^. The list of mutations associated with resistance to first-line antituberculosis drugs in clustered isolates showed that their transmission is likely to occur at the MDR-TB level. Among the first-line antituberculosis drugs, the greatest mutational variability in clustered isolates was demonstrated in the *embB* (encoding resistance to ethambutol) and *pncA* (encoding resistance to pyrazinamide) genes. In addition, mutations in the *pncA* gene were also unique in most of unclustered isolates. Similar diversity has been confirmed in other studies, supporting the theory that *pncA* mutations still remain the leading cause of pyrazinamide resistance^47,48^. Also, 4 isolates integrated within cluster 1 harboured an additional mutation in the *rpoC* gene. In contrast, a detailed look at mutations encoding resistance to second-line antituberculosis drugs (predominantly mutations in *gyrA* gene that confer resistance to fluoroquinolones and *rrs* gene that confer resistance to aminoglycosides) revealed several differences in isolates within the same cluster (cluster 1, 3, 4, 5; Supplementary Tables 1, 2). This indicates that these clustered strains evolved differently due to non-adherence to the treatment regimen, rather than by recent transmission events. Linezolid resistance (mutation *rplC* C154A) was confirmed in 2 isolates within the cluster 3. These findings highlight the need to control linezolid dosing in a resistant TB treatment regimen and support the theory of linezolid resistance association with the Beijing lineage^49,50^.

However, our study has some limitations. Several XDR/MDR cultures could not be reactivated in MGIT medium even after several attempts. In addition, WGS results could not be compared with other genotypic methods. Nevertheless, this study confirmed the utility of WGS in surveillance of drug resistant TB strains circulating in the Czech Republic. Sequencing data analysis identified complete resistance profiles of MDR and XDR strains of *M. tuberculosis*, suggesting that the implementation of this methodology in routine diagnostics could improve TB control at the national level.

## Conclusion

This study highlights the relevance and utility of WGS as a high-resolution approach to perform genetic analysis of drug-resistant TB in the Czech Republic. WGS has detected gene mutations encoding resistance to first-line and second-line antituberculosis drugs (including delamanide and linezolid), demonstrating its clinical importance in TB therapy. In addition, our study provided a baseline genotypic characterization of the MDR- and XDR-TB strains circulating in the Czech Republic and, in combination with epidemiological information, revealed several transmission chains that will improve the surveillance activities in the country.

## Materials and methods

### Sample collection

The culture or sputum smear microscopy positive TB samples used in this study were collected at the National Reference Laboratory for Mycobacteria in Prague, Czech Republic. In total, 65 samples from 2005 to 2020 (representing 60% of all MDR strains) with confirmed phenotypic resistance to at least rifampicin and isoniazid (MDR-TB) were studied. The presence of *M. tuberculosis* in biological material was confirmed by smear positivity and culture positivity on Löwenstein-Jensen (LJ) solid medium and in BACTEC™ MGIT™ 960 System (Becton, Dickinson and Company, East Rutherford, USA). All the work related to the manipulation of live bacteria culture was performed in biosafety level 3 laboratory.

### Drug susceptibility testing

Conventional phenotypic drug susceptibility testing (pDST) by proportion method with critical concentrations of antituberculosis drugs according to WHO recommendations was performed for rifampicin (40.0 μg/ml), isoniazid (0.2 μg/ml), ethambutol (2.0 μg/ml), pyrazinamide (400.0 μg/ml), streptomycin (4.0 μg/ml), kanamycin (30.0 μg/ml), ethionamide (40.0 μg/ml), cycloserine (40.0 μg/ml), paraminosalycylic acid (1.0 μg/ml) and moxifloxacin (1.0 μg/ml) and used as a golden standard to define XDR-TB and MDR-TB^51^. The results were obtained after 3–6 weeks of incubation at 37 °C.

### Whole genome sequencing

1 ml of early positive MGIT cultures were inactivated at 95 °C for 30 min in the biosafety level 3 laboratory. The genomic DNA from inactivated bacterial cultures was extracted according to the manufacturer’s protocol using QIAamp DNA Mini Kit (QIAGEN, Hilden, Germany). DNA concentration was quantified by Qubit 4 technology using Qubit dsDNA HS Assay kit (Thermo Fisher Scientific, Waltham, USA). A concentration of genomic DNA in the range of 0.2–0.4 ng was used to prepare sequencing libraries using the Illumina Nextera XT library preparation kit (Illumina, San Diego, USA). Whole genome sequencing was executed on the Illumina MiSeq platform (Illumina, San Diego, USA) producing paired-end FASTQ files.

### Bioinformatics analysis

Paried-end Illumina read data was first prepared for analysis in the following manner: we used trimmomatic (v0.39) to remove any adapter fragments and low-quality tail-ends with quality phred-scores ≤ Q3^52^. All reads shorter than 35 bp in length were discarded. We then used Kraken2 (v2.2.1) to screen libraries for any contaminating reads that were assigned to taxa outside of the *Mycobacterium tuberculosis* complex using a custom database^53^. The remaining reads were quality-assessed using seqkit^3^ (v0.15)^54^. To assign isolates to MTBC lineages, and identify antibiotic resistance markers (SNPs, indels and frameshifts), we used TB-profiler (v2.8.6; using the *M. tuberculosis* H37Rv genome as the mapping reference)^29^. We identified one isolate (CZ445-15) that consisted of a mix of two lineages, which was excluded from the rest of the analyses.

For phylogenetic and cluster analysis we identified SNPs in the following manner: Reads were mapped against the hypothetical MTBC ancestor reference genome, described previously, using BWA (v0.7.17), and de-duplicated, to remove optical PCR duplicate reads, using picard tools (v2.12; https://broadinstitute.github.io/picard/)^55^. We then used samtools to identify fixed variants with a frequency of at least 70% and removed any variant identified within repetitive genomic regions (PE/PPE), insertion sequences, as well as prophages. A concatenated alignment of 3657 identified SNP positions was then generated from the resulting vcf-files which was used to generate a distance matrix of SNP distances using the dna.dist function implemented in the R package ape^56^. We used IQ-TREE (v1.6) to construct a maximum likelihood phylogeny using an GTR + F + ASC evolutionary model. The IQ-tree software searches through several evolutionary models and selects for a “best-fit model” using the Bayesian Information Criterion (BIC) score assigned to each tested model. The GTR + F + ASC model consistently scored the highest.

We then used the iTol web-based phylogeny tool (v5) to visualize the tree^57,58^. A distant matrix was generated from MTBseq and a minimum spanning tree was constructed using GrapeTree software (https://github.com/achtman-lab/GrapeTree) with the maximum distance threshold of 12 SNPs (due to reporting an analysis of strains collected in 15 years timeframe) for linked transmission^59,60^.

### Ethics statement

This study was approved by the Ethics committee of Jessenius Faculty of Medicine in Martin (EK 72/2018), Comenius University in Bratislava, Slovakia. All methods were carried out in accordance with relevant guidelines and regulations. Informed consent was obtained from all participants or, if participants are under 18, from a parent and/or legal guardian.

## Supplementary Information

Supplementary Tables.

## Data Availability

WGS raw reads were submitted to the European Nucleotide Archive as FASTQ files under study Accession No. PRJEB48710.
