# Supplementary material for: Whole genome sequencing of multidrug-resistant Mycobacterium tuberculosis isolates collected in the Czech Republic, 2005–2020
Source: Sci Rep. 2022 May 3;12:7149. doi: 10.1038/s41598-022-11287-5 (PMC9062869; doi:10.1038/s41598-022-11287-5)
Supplement: Supplementary file 1 — Supplementary Tables. [file 41598_2022_11287_MOESM1_ESM.pdf]

Whole genome sequencing of multidrug-resistant *Mycobacterium tuberculosis* isolates collected in the Czech Republic, 2005-2020

Matúš Dohál[1*], Věra Dvořáková[2], Miluše Šperková[2], Martina Pinková[2], Andrea Spitaleri[3], Anders Norman[4], Andrea Maurizio Cabibbe[3], Erik Michael Rasmussen[4], Igor Porvazník[5,6], Mária Škereňová[7], Ivan Solovič[5,6], Daniela Maria Cirillo[3], Juraj Mokrý[1]

[1]Department of Pharmacology and Biomedical Centre Martin, Jessenius Faculty of Medicine, Comenius University, Slovakia, [2]National Reference Laboratory for Mycobacteria, National Institute of Public Health, Praha, Czech Republic, [3]Emerging Bacterial Pathogens Unit, IRCCS San Raffaele Scientific Institute, Milan, Italy, [4]International Reference Laboratory of Mycobacteriology, Statens Serum Institut, Copenhagen, Denmark, [5]National Institute of Tuberculosis, Lung Diseases and Thoracic Surgery, Vyšné Hágy, Slovakia, Faculty of Health, Catholic University, Ružomberok, Slovakia, [6]Faculty of Health, Catholic University, Ružomberok, Slovakia, [7]Biomedical Center Martin, Department of Molecular Medicine, Jessenius Faculty of Medicine in Martin, Comenius University in Bratislava, Slovakia, Department of Clinical Biochemistry, Jessenius Faculty of Medicine in Martin, Comenius University in Bratislava, Slovakia

*corresponding author – dohal1@uniba.sk

Supplementary table 1. Transmission clusters of MDR, pre-XDR and XDR *M. tuberculosis* isolates from Czech Republic

Cluster (sublineage)	Isolates (year of the first diagnosis of TB)	Resistance profile (determined by WGS)	Location
1 (Haarlem 4.1.2.1)	CZ286 (2008)	MDR	Prague
	CZ188 (2004)	XDR	Prague
	CZ8842 (2014)	MDR	Nový Jičín
	CZ218 (2004)	Pre-XDR	Prague
	CZ188-16 (2016)	MDR	Prague
2 (Beijing 2.2.1)	CZ800-17 (2017)	MDR	Roudnice nad Labem
	CZ388-19 (2014)	MDR	Prague
3 (Beijing 2.2.1)	CZ553-17 (2017)	XDR	Pilsen
	CZ628-17 (2014)	MDR	Prague
	CZ29-19 (2019)	MDR	Pilsen
	CZ149-18 (2014)	MDR	Prague
	CZ139-19 (2019)	Pre-XDR	Prague
4 (Beijing 2.2.1)	CZ291 (2009)	XDR	Kladno
	CZ372-15 (2015)	MDR	Prague
5 (Beijing 2.2.1)	CZ279 (2008)	XDR	Šenov
	CZ276 (2007)	MDR	Liberec
	CZ178-18 (2017)	MDR	Šenov
6 (Beijing 2.2.1)	779-16 (2016)	MDR	Prague
	1019-16 (2016)	MDR	Mělník

Supplementary table 2. Study data and drug resistance related mutations in candidate genes

Cluster	Sample	Year	Sex	Age		Rifampicin	Izoniazid	Ethambutol	Pyrazinamide	Streptomycin	Aminoglycosides	Ethionamide	Fluoroquinolones	PAS	Linezolid	Delamanid
1	188/16	2016	M	53	Euro-American (Haarlem) 4.1.2.1	*rpoB* p. Ser450Leu	*katG* p.Ser315Thr	*embB* p.Met306Leu		*rrs* r.513c<t		*ethA* c.1054_1054del				
1	8842	2014	M	49	Euro-American (Haarlem) 4.1.2.1	*rpoB* p. Ser450Leu, *rpoC* p.Leu527Val	*katG* p.Ser315Thr	*embB* p.Met306Leu		*rrs* r.514a>c		*ethA* c.1054_1054del				
1	188	2005	F	56	Euro-American (Haarlem) 4.1.2.1	*rpoB* p. Ser450Leu, *rpoC* p.Leu527Val	*katG* p.Ser315Thr	*embB* p.Met306Leu		*rrs* r.906a>g	*rrs* r.1401a>g	*ethA* c.1054_1054del	*gyrA* p.Ala90Val			
1	286	2008	M	70	Euro-American (Haarlem) 4.1.2.1	*rpoB* p. Ser450Leu, *rpoC* p.Leu527Val	*katG* p.Ser315Thr	*embB* p.Met306Leu		*rrs* r.906a>g		*ethA* c.1054_1054del				
1	218	2005	M	54	Euro-American (Haarlem) 4.1.2.1	*rpoB* p. Ser450Leu, *rpoC* p.Leu527Val	*katG* p.Ser315Thr	*embB* p.Met306Leu		*rrs* r.514a>c		*ethA* c.1054_1054del	*gyrA* p.Asp94Gly			
2	800/17	2017	M	32	East-Asian (Beijing) 2.2.1	*rpoB* p. Ser450Leu	*katG* p.Ser315Thr	*embB* p.Met306Val	*pncA* p.Thr160Ala	*rpsL* p.Lys43Arg						
2	388/19	2019	M	38	East-Asian (Beijing) 2.2.1	*rpoB* p. Ser450Leu	*katG* p.Ser315Thr	*embB* _p.Met306Val	*pncA* p.Thr160Ala	*rpsL* p.Lys43Arg						
3	149/18	2018	M	37	East-Asian (Beijing) 2.2.1	*rpoB* p. Ser450Leu	*katG* p.Ser315Thr		*pncA* c.405_406insCACC	*rpsL* p.Lys43Arg					*rplC* p.Cys154Arg	
3	29/19	2019	M	53	East-Asian (Beijing) 2.2.1	*rpoB* p. Ser450Leu	*katG* p.Ser315Thr	*embA* c.-8C>A (only PhyResSe)		*rpsL* p.Lys43Arg						
3	139/19	2019	F	26	East-Asian (Beijing) 2.2.1	*rpoB* p. Ser450Leu	*katG* p.Ser315Thr	*embB* p.Gln497Arg	*pncA* c.405_406insCACC	*rpsL* p.Lys43Arg			*gyrA* p.Asp94Gly, *gyrA* p.Ala90Val		*rplC* p.Cys154Arg	
3	628/17	2017	M	36	East-Asian (Beijing) 2.2.1	*rpoB* p. Ser450Leu	*katG* p.Ser315Thr		*pncA* c.405_406insCACC	*rpsL* p.Lys43Arg						
3	553/17	2017	F	29	East-Asian (Beijing) 2.2.1	*rpoB* p. Ser450Leu	*katG* p.Ser315Thr		*pncA* p.Thr160Pro	*rpsL* p.Lys43Arg	*rrs* r.1401a>g		*gyrA* p.Asp94Gly			
4	372/15	2015	M	43	East-Asian (Beijing) 2.2.1	*rpoB* p. Ser450Leu	*katG* p.Ser315Thr	*embB* p.Gly406Ala	*pncA* p.Ile31Ser	*rpsL* p.Lys43Arg		*ethA* c.341_341del				
4	291	2009	F	49	East-Asian (Beijing) 2.2.1	*rpoB* p. Ser450Leu	*katG* p.Ser315Thr	*embB* p.Gly406Ala	*pncA* p.Ile31Ser	*rpsL* p.Lys43Arg	*rrs* r.1401a>g	*ethA* c.341_341del	*gyrA* p.Asp94Gly			
5	178/18	2018	M	51	East-Asian (Beijing) 2.2.1	*rpoB* p. Ser450Leu	*katG* p.Ser315Thr	*embB* p.Gln497Arg		*rpsL* p.Lys43Arg				*folC* p.Glu40Gly		
5	279	2009	M	41	East-Asian (Beijing) 2.2.1	*rpoB* p. Ser450Leu	*katG* p.Ser315Thr	*embB* p.Gln497Arg		*rpsL* p.Lys43Arg			*gyrA* p.Ala90Val			*ddn* p. Trp88STOP
5	276	2010	M	54	East-Asian (Beijing) 2.2.1	*rpoB* p. Ser450Leu	*katG* p.Ser315Thr	*embB* p.Gln497Arg		*rpsL* p.Lys43Arg				*folC* p.Glu40Gly		
6	1019/16	2016	M	81	East-Asian (Beijing) 2.2.1	*rpoB* p. Ser450Leu	*katG* p.Ser315Thr	*embB* p.Met306Ile		*rpsL* p.Lys43Arg						
6	779/16	2016	M	49	East-Asian (Beijing) 2.2.1	*rpoB* p. Ser450Leu	*katG* p.Ser315Thr	*embB* p.Met306Ile		*rpsL* p.Lys43Arg						
-	772/17	2017	M	49	East-Asian (Beijing) 2.2.1	*rpoB* p. Ser450Leu	*katG* p.Ser315Thr	*embB* p.Met306Ile	*pncA* p.Thr142Ala	*rpsL* p.Lys43Arg	*rrs* r.1401a>g					
-	851/17	2017	M	48	East-Asian (Beijing) 2.2.1	*rpoB* p. Ser450Leu	*katG* p.Ser315Thr. *fabG1* c.-15C>T	*embA* c.-16C>T, *embB* p.Pro404Ser	*pncA* p.Gln10Lys	*rpsL p.* Lys88Arg	*rrs* r.1401a>g	*fabG1* c.-15C>T	*gyrA* p.Asp94Gly	*folC* p.Asp49Phe		
-	863/17	2017	F	50	East-Asian (Beijing) 2.2.1	*rpoB* p. Ser450Leu	*katG* p.Ser315Thr. *fabG1* c.-15C>T	*embA* c.-12C>T, *embB* p.Tys334His	*pncA* p.His71Arg	*rpsL p.* Lys88Arg		*fabG1* c.-15C>T	*gyrA* p.Ala90Val			
-	395/18	2018	M	27	East-Asian (Beijing) 2.2.1	*rpoB* p. Ser450Leu	*katG* p.Ser315Thr	*embB* p.Met306Val	*pncA* p.His51Arg	*rpsL* p.Lys43Arg						
-	412/18	2018	M	29	Euro-American (Haarlem) 4.1.2.1	*rpoB* c.1302_1307del	*katG* p.Ser315Thr									
-	424/18	2018	M	28	Euro-American (LAM, T) 4.3.3	*rpoB* p.His445Leu	*katG* p.Ser315Thr. *fabG1* c.-15C>T	*embB* p.Met306Ile	*pncA* p.Cys138Tyr	*rrs* r.514a>c		*fabG1* c.-15C>T				
-	505/18	2018	M	38	Euro-American (LAM, T) 4.3.4.2	*rpoB* p. Ser450Leu	*katG* p.Ser315Thr	*embB* p.Met306Ile					*gyrA* p.Asp94Asn			
-	279/19	2019	F	31	East-Asian (Beijing) 2.2.1	*rpoB* p. Ser450Leu	*katG* p.Ser315Thr	*embB* p.Asp354Ala	*pncA* p.Asp63Ala	*rpsL* p.Lys43Arg	*rrs* r.1401a>g		*gyrA* p.Asp94Gly			*ddn* p.Trp88STOP
-	318/19	2019	M	39	Euro-American (LAM, T) 4.3.3	*rpoB* p. Ser450Trp	*katG* p.Ser315Thr. *fabG1* c.-15C>T			*rrs* r.514a>c		*fabG1* c.-15C>T				
-	354/19	2019	M	40	East-Asian (Beijing) 2.2.1	*rpoB* p. Ser450Leu	*katG* p.Ser315Thr. *fabG1* c.-15C>T	*embA* c.-12C>T, *embB* p.Tys334His	*pncA* p.His57Arg	*rpsL p.* Lys88Arg		*fabG1* c.-15C>T				
-	389/19	2019	M	26	East-Asian (Beijing) 2.2.1	*rpoB* p. Ser450Phe	*katG* p.Ser315Thr	*embB* p.Met306Val	*pncA* p.Leu27Pro	*rpsL* p.Lys43Arg	*rrs* r.1401a>g		*gyrA* p.Asp94Gly			
-	402/19	2019	M	46	East-Asian (Beijing) 2.2.1	*rpoB* p. Ser450Leu	*katG* p.Ser315Thr	*embB* p.Tyr319Cys	*pncA* p.Asp63Ala	*rpsL* p.Lys43Arg	Kan. e*is* c.-14C>T					
-	532/18	2018	M	45	Euro-American (LAM, T) 4.3.3	*rpoB* p. Ser450Leu	*katG* p.Ser315Thr	*embB* p.Gly406Asp		*rpsL* p.Lys43Arg						
-	541/18	2018	M	33	East-Asian (Beijing) 2.2.1	*rpoB* p. Ser450Leu	*katG* p.Ser315Thr. *fabG1* c.-15C>T	*embA* c.-12C>T, *embB* p.Tys334His		*rpsL* p.Lys88Arg		*fabG1* c.-15C>T				
-	614/18	2018	M	38	East-Asian (Beijing) 2.2.1	*rpoB* p.Leu452Pro	*katG* p.Ser315Thr	*embB* p.Met306Val	*pncA* p.His51Arg	*rpsL* p.Lys43Arg	Kan. e*is* c.-12C>T	*ethA* c.110_110del				
-	757/18	2018	M	28	Euro-American (Haarlem) 4.1.2.1	*rpoB* p. Ser450Leu, *rpoC* p.Leu527Val	*katG* p.Ser315Thr	*embB* p.Met306Val	*pncA* p.Phe94Leu			*ethA* c.1054_1054del				
-	35/19	2019	M	53	East-Asian (Beijing) 2.2.1	*rpoB* p. Ser450Trp	*katG* p.Ser315Thr	*embB* p.Tyr319Ser		*rpsL* p.Lys43Arg	*rrs* r.1401a>g					
-	36/19	2019	M	41	East-Asian (Beijing) 2.2.1	*rpoB* p. Ser450Leu	*katG* p.Ser315Thr	*embB* p.Met306Val	*pncA* p.Pro69Leu	*rpsL* p.Lys43Arg		*ethA* p.Thr314Ile	*gyrB* p.Thr500Pro			
-	105/19	2019	M	55	East-Asian (Beijing) 2.2.1	*rpoB* p. Ser450Leu, *rpoC* p.Phe452Ser	*katG* p.Ser315Thr	*embB* p.Met306Val	*pncA* p.His57Arg	*rpsL* p.Lys43Arg	Kan. *eis* c.-10G>A	*ethA* p.Met1Arg	*gyrA* p.Asp94Gly			
-	732/19	2019	M	30	Euro-American (Ural) 4.2.1	*rpoB* p. Ser450Leu	*katG* p.Ser315Thr. *fabG1* c.-15C>T	*embB* p.Ser297Ala		*rpsL p.* Lys88Arg	Kan. e*is* c.-12C>T	*fabG1* c.-15C>T				
-	19/15	2015	M	38	Euro-American (LAM, T) 4.3.3	*rpoB* p. Ser450Leu	*katG* p.Ser315Thr. *fabG1* c.-15C>T	*embB* p.Gln497Arg	*pncA* p.Asp63Gly	*rrs* r.514a>c		*fabG1* c.-15C>T	*gyrA* p.Asp94Asn			
-	416/15	2015	F	38	East-Asian (Beijing) 2.2.1	*rpoB* p. Ser450Leu	*katG* p.Ser315Thr	*embB* p.Gln497Arg	*pncA* p.Val139Ala	*rpsL* p.Lys43Arg						
-	367/17	2017	M	52	Euro-American (Haarlem) 4.1.2.1	*rpoB* p. Ser450Leu, *rpoC* p.Leu527Val	*katG* p.Ser315Thr	*embB* p.Met306Leu	*pncA* p.Phe94Leu			*ethA* c.1054_1054del				
-	398/15	2015	M	30	East-Asian (Beijing) 2.2.1	*rpoB* p. Ser450Leu	*katG* p.Ser315Thr	*embB* p.Gly406Ser	*pncA* p.Gln10Pro, *pncA* c.287_287del	*rpsL* p.Lys43Arg		*ethA* p.Arg67Pro	*gyrA* p.Asp94Asn, *gyrA* p.Ser91Pro			
-	270/20	2020	M	32	East-Asian (Beijing) 2.2.1	*rpoB* p. Ser450Leu, *rpoC* p.Phe452Ser	*katG* p.Ser315Thr	*embB* p.Met306Leu	*pncA* p.His57Arg	*rpsL* p.Lys43Arg	Kan. *eis* c.-10G>A	*ethA* p.Met1Arg	*gyrA* p.Ser91Pro			
-	421/17	2017	M	50	East-Asian (Beijing) 2.2.1	*rpoB* p. Ser450Leu	*katG* p.Ser315Thr	*embB* p.Gln497Arg	*pncA* p.Val139Ala							
-	318/15	2015	F	28	East-Asian (Beijing) 2.2.1	*rpoB* p. Ser450Leu, *rpoC* o.Ile491Thr	*katG* p.Ser315Thr	*embB* p.Met306Val	*pncA* c.524_524del	*rpsL* p.Lys43Arg	Kan. e*is* c.-10G>A (low-level R)	*ethA* c.1010_1010del				
-	505/15	2015	M	23	East-Asian (Beijing) 2.2.1	*rpoB* p. Ser450Leu	*katG* p.Ser315Thr			*rpsL* p.Lys43Arg						
-	9320	2014	F	29	East-Asian (Beijing) 2.2.1	*rpoB* p. Ser450Leu	*katG* p.Ser315Thr	*embB* p.Met306Ile		*rpsL* p.Lys43Arg						
-	8946	2014	F	30	Euro-American (LAM, T) 4.3.3	*rpoB* p. Ser450Leu	*katG* p.Ser315Thr. *fabG1* c.-15C>T	*embB* p.Met306Val	*pncA* p.Asp12Ala	*rrs* r.514a>c	Kan. *eis* c.-10G>C, Capreo. *tlyA* c.733_742del	*fabG1* c.-15C>T				
-	445/15	2015	M	43	Euro-American (Haarlem) 4.1.2.1	*rpoB* p.Asp435Tyr	*katG* p.Ser315Thr. *fabG1* c.-15C>T	*embB* p.Met306Ile				*fabG1* c.-15C>T				
-	95/17	2017	M	32	East-Asian (Beijing) 2.2.1	*rpoB* p. Ser450Leu	*katG* p.Ser315Thr	*embB* p.Met306Val		*rpsL* p.Lys43Arg						
-	248/20	2009	M	34	East-Asian (Beijing) 2.2.1	*rpoB* p.Asp435Tyr	*katG* p.Ser315Thr	*embB* p.Met306Ile	*pncA* Ile133Thr	*rpsL* p.Lys43Arg						
-	9206	2014	F	27	East-Asian (Beijing) 2.2.1	*rpoB* p. Ser450Leu	*katG* p.Ser315Thr	*embB* p.Met306Val	*pncA* p.Val128Gly	*rpsL* p.Lys43Arg						
-	317/15	2015	M	39	East-Asian (Beijing) 2.2.1	*rpoB* p.Asp435Tyr, *rpoB* p. Ser450Leu	*inhA* p.Ser94Ala, *fabG1* c.-15C>T	*embB* p.Met306Val	*pncA* p.Asp12Glu	*rpsL* p.Lys43Arg		*inhA* p.Ser94Ala, *fabG1* c.-15C>T	*gyrA* p.Asp94Gly			
-	895/16	2016	M	29	East-Asian (Beijing) 2.2.1	*rpoB* p.Ser450Trp	*katG* p.Ser315Thr	*embB* p.Met306Val		*rpsL* p.Lys43Arg						
-	417/15	2015	M	47	East-Asian (Beijing) 2.2.1	*rpoB* p. Ser450Leu	*katG* p.Ser315Thr	*embB* p.Met306Ile		*rpsL* p.Lys43Arg	*rrs* r.1401a>g	*ethA* c.32_32del	*gyrA* p.Asp94Gly			
-	292	2009	M	28	East-Asian (Beijing) 2.2.1	*rpoB* p.His445Asp	*katG* p.Ser315Thr, *fabG* c.-8T>C	*embB* p.Met306Ile				fabG c.-8T>C				
-	217	2005	M	37	Euro-American (Ural) 4.2.1	*rpoB* p. Ser450Leu	*katG* p.Ser315Thr. *fabG1* c.-15C>T	*embB* p.Gln497Arg		*rpsL p.* Lys88Arg	Kan. e*is* c.-12C>T	*fabG1* c.-15C>T				
-	223	2006	F	51	Euro-American (Ural) 4.2.1	*rpoB* p. Ser450Leu	*katG* p.Ser315Thr. *fabG1* c.-15C>T	*embB* p.Gly406Ala	*pncA* p.Tyr34STOP	*rpsL p.* Lys88Arg	Kan. e*is* c.-12C>T	*fabG1* c.-15C>T				
-	280	2008	M	53	Euro-American (LAM) 4.3.1	*rpoB* p.Asp435Val	*katG* p.Ser315Thr									
-	221	2009	M	28	East-Asian (Beijing) 2.2.1	*rpoB* p. Ser450Leu, *rpoC* p.Ile491Thr	*katG* p.Ser315Thr, *fabG* c.-8T>C	*embB* p.Met306Ile	*pncA* p.Trp68Gly	*rpsL* p.Lys43Arg		*ethA* c.1386_1386del, *fabG* c.-8T>C				
-	297	2010	F	23	East-Asian (Beijing) 2.2.1	*rpoB* p. Ser450Leu	*katG* p.Ser315Thr	*embB* p.Met306Ile	*pncA* p.Leu19Pro	*rpsL* p.Lys43Arg	Kan. e*is* c.-12C>T					

-	222	2008	M	33	Euro-American (LAM, T) 4.3.3	*rpoB* p.His445Asp	*katG* p.Ser315Thr, *fabG1* c.-15C>T	*embA* c.-12C>T, *embB* p.Gln497Arg	*pncA* p.Asp49Gly	*rrs*	Kan. e*is* c.-12C>T	*fabG* 1 c.-15C>T				
-	288	2009	M	49	East-Asian (Beijing) 2.2.1	*rpoB* p. Ser450Leu, *rpoC* p.Ile491Thr	*katG* p.Ser315Thr	*embB* p.Met306Val	*pncA* p.Gly97Arg	*rpsL* p.Lys43Arg						
-	282	2009	M	25	East-Asian (Beijing) 2.2.1	*rpoB* p. Ser450Leu	*katG* p.Ser315Thr	*embB* p.Met306Val		*rpsL* p.Lys43Arg						
